# *Chenopodium ambrosioides* associated with whole body vibration exercises alters the feed intake in Wistar rats

**DOI:** 10.1042/BSR20170846

**Published:** 2017-08-21

**Authors:** André Luiz Bandeira Dionizio Cardoso, Éric Heleno Freire Ferreira Frederico, Carlos Alberto Sampaio Guimarães, Lívia Pinto Almeida, Rosane de Figueiredo Neves, Danúbia Cunha de Sá-Caputo, Eloá Moreira-Marconi, Carla de Fontoura Dionello, Danielle Soares Morel, Laisa Liane Paineiras-Domingos, Cintia Renata Sousa-Gonçalves, Nasser Ribeiro Asad, Mario Bernardo-Filho

**Affiliations:** 1 Programa de Pós-Graduação em Ciências Médicas, Faculdade de Ciências Médicas, Universidade do Estado do Rio de Janeiro, Rio de Janeiro 20551-030, RJ, Brasil; 2 Laboratório de Vibrações Mecânicas e Práticas Integrativas e Complementares, Departamento de Biofísica e Biometria, Instituto de Biologia Roberto Alcantara Gomes, Universidade do Estado do Rio de Janeiro, Rio de Janeiro 20551-030, RJ, Brasil; 3 Programa de Pós-graduação em Biociências, Instituto de Biologia Roberto Alcantara Gomes, Universidade do Estado do Rio de Janeiro, Rio de Janeiro 20551-030, RJ, Brasil; 4 Programa de Pós-Graduação em Fisiopatologia Clínica e Experimental, Faculdade de Ciências Médicas, Universidade do Estado do Rio de Janeiro, Rio de Janeiro 20551-030, RJ, Brasil

**Keywords:** Chenopodium ambrosioides, body mass, enzymatic activity, feed intake, physiological parameters, whole body vibration

## Abstract

The consequences of treatment involving the use of a natural product and whole body vibration (WBV) exercise have been investigated. The aim of the present study was to evaluate the effects of the joint treatment with an aqueous extract of *Chenopodium ambrosioides* and WBV on physiological parameters in rats. Wistar rats (*n*=20) were divided equally into four groups: control group (CG), treated with *C. ambrosioides* (CHE) group, exposed to 50 Hz of mechanial vibration (VBR), and treated with *C. ambrosioides* and exposed to 50 Hz of mechanical vibration (VBR + CHE) daily for 6 weeks. The body mass of the animals was determined weekly, the feed intake and the stool consistency were measured daily. One day after the 6 weeks of treatment, samples of blood were collected and used for biochemical analysis. Along 6 weeks, there was an increase (*P*<0.001) in the feed intake in VBR group and a decrease in the CHE group in comparison with other groups. The levels of the enzyme aspartate aminotransferase (AST) in VBR + CHE group decreased (*P*<0.05) in comparison with other groups. No differences were found in body mass and stool consistency. WBV altered the feed intake without directly affecting the body mass. Moreover, WBV in association with *C. ambrosioides* caused alteration in the enzymatic activity of AST.

## Introduction

Many plants have been particularly used in the discovery and development of new drugs for the treatment of many clinical situations. The search for scientific evidence has invigorated validation studies of numerous plant species [1,2]. *Chenopodium ambrosioides* is an example of natural product used in the folk medicine as an antimicrobial, healing, and anti-inflammatory agent [3,4]. Belonging to the Chenopodiaceae family, this plant is a herbaceous shrub native to Central and South America. In Brazil, it is known as ‘Mastruz’ or ‘erva-de-Santa-Maria’ [5,6]. Some investigations have demonstrated the anti-inflammatory [7], antihelmintic [8], antitumoral [9] and antibacterial [10] effects of *C. ambrosioides*.

Studies have shown the effect of treatments involving natural products associated with mechanical vibrations [11–13]. Mechanical vibration can be described as a physical stimulus characterized by an oscillatory motion about an equilibrium point. It can be defined by biomechanical parameters, such as frequency, peak-to-peak displacement, and peak acceleration [14,15]. Devices able to generate this vibration as oscillating/vibratory platform (OVP) have been suggested as an alternative to promote physical improvement in athletes, health-compromised individuals, and the elderly [16].

Mechanical vibrations produced by OVP can be transmitted to the whole body; when an individual is on the base of the platform that is turned on, generating whole body vibration (WBV) exercise [17–19]. WBV is well known as an alternative exercise modality for enhancing balance and flexibility [20], muscle activity, force and power [21–23], and improvement in bone mineral density [24,25]. Some authors [15,26,27] have discussed that the effects of the WBV exercises would be related to alteration on the plasma concentration of biochemical markers.

The aim of the present study was to evaluate the effects of an aqueous extract of *C. ambrosioides* in rats simultaneously submitted to WBV exercises (50 Hz) on body mass, feed intake, stool consistency, and biochemical analysis. Although, it is unknown whether mechanical vibration associated with the extract of *C. ambrosioides* has the potential to induce long-term changes in factors related with physiological activity, it is expected to have alterations in some of the analyzed parameters due to this simultaneous treatment.

## Materials and methods

### Animals and ethical approach

Adult male Wistar rats, approximately 3–4 months (between 250 and 300 g) were used. The animals were kept under care (25 ± 2°C, 12 h of light/dark cycle) and fed with a standard diet and water *ad libitum*. All experiments were conducted following the standards of the Comitê de Ética Para o Uso de Animais Exprimentais (CEUA), Instituto de Biologia Roberto Alcantara Gomes, Universidade do Estado do Rio de Janeiro, Brazil, approved with the registration number: CEUA/041/2013.

### Plant material

A commercial dry extract of *C. ambrosioides* (Chenopodiaceae) was used (lot: 032015, validity up to July 2016, Chá & Cia – Ervas Medicinais, São José dos Campos, SP, Brazil). The dose was chosen according to previously described by Pereira et al. [28] with slight modification. To prepare the extract, 300 mg of *C. ambrosioides* was added to 20 ml of deionized water (Permution, E.J Krieger & Cia Ltda, Curitiba, PR, Brazil) at 100°C. This preparation remained at rest for 10 min. After this period of time, it was filtered (commercial paper filter, lot: 103/1 E6 11:26, validity up to March 2017, Melitta, São Paulo, Brazil) and the supernatant was considered to be 15 mg/ml.

### Spectrophotometry of *C. ambrosioides* extract

The absorbance spectrum (spectrophotometer, Analyser Comércio e Indústria Ltda., São Paulo, Brazil) was determined (400–700 nm) with *C. ambrosioides* (15 mg/ml). Saline solution (0.9% NaCl) was used as the blank. The absorbance was measured at each interval of 10 nm wavelength. The value of the absorbance was maximum (0.160 ± 0.010) at 440 nm with *C. ambrosioides* (mastruz) extract of 15 mg/ml. This value was considered as the marker to control the quality of the preparation of the extract.

### Experimental design

Wistar rats (*n*=20) were divided into four groups: control (CG), treated with *C. ambrosioides* (CHE), exposed to 50 Hz mechanical vibration (VBR), and treated with *C. ambrosioides* and exposed to 50 Hz mechanical vibration (VBR + CHE). The CG received by gavage [29] 1.0 ml of deionized water, while the group CHE received the same via 1.0 ml of aqueous extract at 15 mg/ml. The group VBR received 1.0 ml of deionized water and group VBR + CHE received 1.0 ml of aqueous extract at 15 mg/ml and submitted to the vibration generated in the platform.

**Figure 1:**
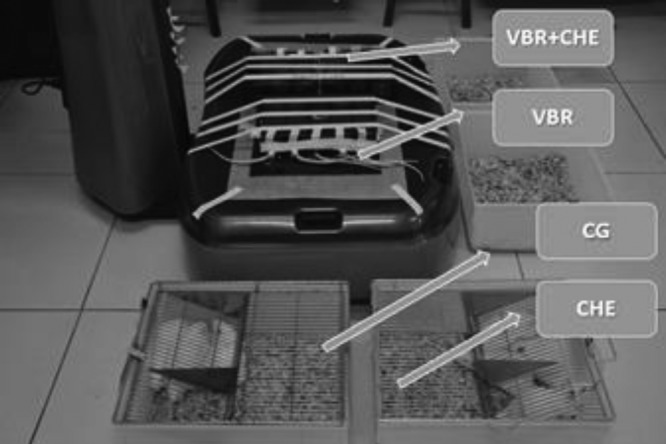
Wistar rats from CG, CHE, VBR, VBR + CHE

For mechanical vibration treatment, animals from VBR and VBR + CHE groups were placed in a box with individual compartments under a platform (Globus-Vibe 800, Italy). Following Pawlak et al. [30] with slight adjustments, the animals in these groups received 5 min of WBV daily for 6 weeks. The treatment consisted of four sessions of 30 s each under the base of the platform at a frequency of 50 Hz, amplitude of 0.78 mm, and peak acceleration of 7.84×***g***, given a 1-min rest period in each session. The animals in CG and CHE groups remained in their cages, which were placed close the platform turned on (Figure 1).

### Body mass analysis

The mass of all the animals was determined weekly on a digital balance (Filizola BP6, São Paulo, Brazil). The relation between the mass of the animals each week in comparison with the first day of this investigation was determined. The value was multiplied by 100.

### Feed intake analysis

Feed intake was measured daily in each group (cage). Five hundred grams of feed was offered daily. On the next day, the left feed was determined on a digital balance (Filizola BP6, São Paulo, Brazil). The consumed feed was calculated by the difference between 500 g and the left feed on each day. Following this, the quantity of feed was completed daily to 500 g.

### Stool consistency analysis

The stool consistency was evaluated with stool form scale adapted by Frederico et al. [11] for Wistar rats. It was considered type 1 (hard and dry stools), type 2 (normal), type 3 (smooth and soft), and type 4 (fluffy pieces with ragged edges). Three different and independent evaluators evaluated the consistency of the stools and the average of these three analyses was considered.

### Blood biochemical analysis

After 6 weeks of experiment, blood samples were collected by cardiac puncture and the concentrations of selected biomarkers (glucose, creatinine, cholesterol, triglyceride, high-density lipoprotein (HDL), alkaline phosphatase, bilirubin, calcium, magnesium, total protein, and albumin) were measured. The concentrations of some enzymes (amylase, lipase, creatine kinase (CK), alanine aminotransferase (ALT), and aspartate aminotransferase (AST)) were also measured. The determination was carried out in the Clinical laboratory of the Hospital Universitário Pedro Ernesto, Universidade do Estado do Rio de Janeiro. The determinations were performed in automated equipment (COBAS INTEGRA 400 plus, Roche, Basel, Switzerland).

### Statistical analysis

As all the studied data did not follow a normal distribution, Kruskal–Wallis test following the post-test Student–Newman–Keuls was done, for the statistical analysis of the results with BioEstat 5.3 (Instituto Mamiraua, Pará, Brasil). Data were presented as mean ± S.D., median ± interquartile range (IQR) or as percentage (%). Statistical significance was accepted at *P*<0.05. Epsilon-squared (ɛ^2^) was analyzed to estimate the effect size [31].

## Results

### The absorbance spectrum of the *C. ambrosioides*

**Figure 2:**
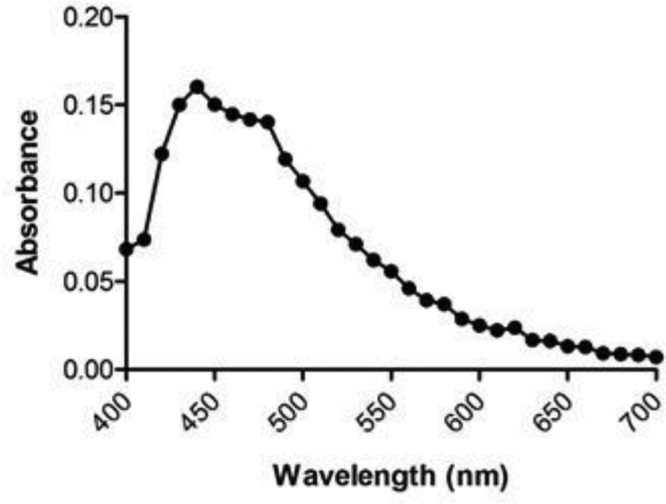
Spectrophotometry of *C. ambrosioides* aqueous extract at 15 mg/ml

Figure 2 shows the absorbance spectrum of *C. ambrosioides* extract at the highest concentration used (15 mg/ml) in the range 400–700 nm. The data showed an absorption peak of the extract with the optical density of 0.160 ± 0.010 at 440 nm. It was used as a marker of the reproducibility of the extract preparation.

### Effect of WBV and/or *C. ambrosioides* on body mass

The initial body mass of the animals of all the groups was considered being 100%. Significant differences in the percentage of body mass were not detected in the animals submitted to the different treatments (Table 1). However, percentages of the mass of the animals of the VBR and VBR + CHE groups were slightly less than the animals of the CG or CHE groups. The values of *ɛ*^2^ ranged from 0 to 0.1586, indicating no effect size.

**Table 1:** Body mass (%) of the groups of animals submitted to different treatments

Week	CG (%)	CHE (%)	VBR (%)	VBR + CHE (%)	*P*	*ɛ*^2^
0	100.00 ± 0.00	100.00 ± 0.00	100.00 ± 0.00	100.00 ± 0.00	0.0000	0.0000
2	102.60 ± 4.96	102.44 ± 6.71	98.98 ± 2.61	100.32 ± 3.31	0.8315	0.0460
3	105.67 ± 4.50	106.03 ± 6.30	101.55 ± 2.60	102.25 ± 3.20	0.6007	0.0982
4	109.02 ± 4.89	109.52 ± 8.35	104.92 ± 1.01	104.60 ± 5.53	0.7302	0.0682
5	111.93 ± 4.91	112.46 ± 6.92	108.48 ± 2.17	105.72 ± 5.35	0.3896	0.1586
6	115.27 ± 4.40	113.72 ± 6.93	110.84 ± 2.04	109.03 ± 7.11	0.6077	0.0965

‘0’ was the first measurement of the mass of the animals. Values are shown as the median ± S.D., *P, ɛ*^2^.

### Effect of WBV and/or *C. ambrosioides* on feed intake

**Figure 3:**
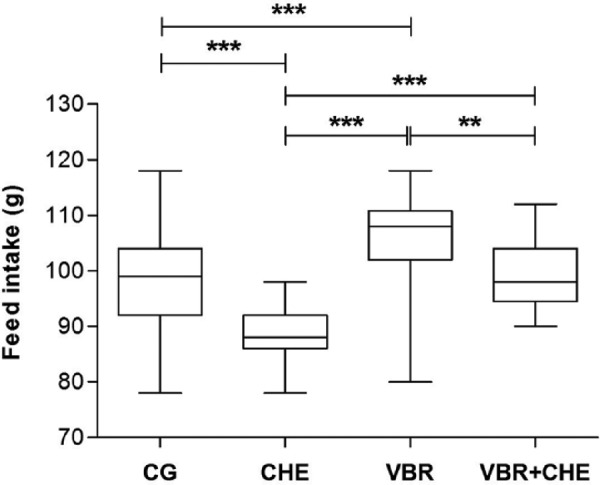
Effects of WBV on feed intake Values are mean ± S.D.; **P<0.001, ***P<0.0001.

The VBR group had a higher (*P*<0.001) feed intake when compared with the other groups, whereas the CHE group had a lower consumption (*P*<0.001) in relation to the other groups. No differences were found in CG and VBR + CHE groups as it is shown in Figure 3.

### Effect of WBV and/or *C. ambrosioides* on stool consistency

In the present study, a stool scale adapted for Wistar rats by Frederico et al. [11] defining four types of stools (hard-1, normal-2, soft middle-3, and pasty-4) was used. The type of feces most identified was type 2. No differences were found in stool consistency amongst the four groups (Table 2). The *ɛ*^2^ was 0.0766 indicating a small relationship.

**Table 2:** Stool consistency of animals submitted to different treatments following a Frederico scale adapted for Wistar rats

Day	CG	CHE	VBR	VBR + CHE	*P*	*ɛ*^2^
1–10	2.00 ± 0.67	2.00 ± 0.42	2.00 ± 0.47	2.00 ± 0.47	0.9835	0.0085
11–20	2.00 ± 0.11	2.00 ± 0.32	2.00 ± 0.58	2.00 ± 0.52	0.1485	0.2810
21–30	2.00 ± 0.00	2.00 ± 0.19	2.00 ± 0.44	2.00 ± 0.16	0.1000	0.0000
31–40	2.00 ± 0.00	2.00 ± 0.00	2.00 ± 0.14	2.00 ± 0.00	>0.05	0.0000

Values are shown as the median ± S.D., *P, ɛ*^2^.

### Effect of WBV and/or *C. ambrosioides* on a general biochemical analysis

After 42 days of treatment, no differences were observed in the plasma concentration of the evaluated biomarkers amongst the animals submitted to the various treatments (Table 3).

**Table 3:** Plasma concentration of some biomarkers in animals submitted to different treatments

Biomarkers	CG	CHE	VBR	VBR + CHE	*P*	*ɛ*^2^
Cholesterol (mmol/l)	1.37 ± 0.26	1.15 ± 0.18	1.42 ± 0.19	1.36 ± 0.07	0.1309	0.1882
Triglyceride (mmol/l)	0.76 ± 0.31	0.66 ± 0.26	0.94 ± 0.35	1.00 ± 0.40	0.3109	0.2965
HDL (mmol/l)	1.33 ± 0.18	1.17 ± 0.22	1.34 ± 0.14	1.29 ± 0.07	0.5846	0.1021
Urea (mmol/l)	16.49 ± 1.54	17.92 ± 1.41	17.64 ± 1.15	15.71 ± 2.11	0.1991	0.2449
Creatinine (μmol/l)	37.13 ± 3.54	38.90 ± 4.42	35.36 ± 6.19	33.59 ± 3.54	0.3540	0.1713
Glucose (mmol/l)	7.08 ± 0.11	8.05 ± 0.99	8.37 ± 1.58	6.72 ± 0.72	0.0821	0.3526
Total protein (mg/dl)	63.20 ± 1.90	59.40 ± 1.50	59.80 ± 3.70	51.60 ± 27.90	0.1738	0.2611
Albumin (g/l)	38.40 ± 2.10	33.60 ± 6.30	37.60 ± 3.60	36.00 ± 20.00	0.2944	0.2056
Bilirubin (μmol/l)	0.96 ± 0.51	1.30 ± 0.17	0.82 ± 0.34	0.89 ± 0.51	0.2063	0.2404
Calcium (mmol/l)	2.56 ± 0.14	2.53 ± 0.12	2.60 ± 0.14	2.61 ± 0.08	0.5739	0.1046
Magnesium (mmol/l)	1.23 ± 0.15	1.29 ± 0.14	1.29 ± 0.13	1.21 ± 0.11	0.6106	0.0956

Values are shown as median ± S.D., *P, ɛ*^2^.

### Effect of WBV and/or *C. ambrosioides* on the enzymatic activities

As shown in the Table 4, after 6 weeks of treatment a small but significant (*P*<0.05) decrease in the plasma level of AST in the animals of the groups VBR + CHE in comparison with the animals of the other groups was observed. The *ɛ*^2^ was 0.5129 indicating a moderate effect size to AST. Nevertheless, no significant change was found in the plasma concentrations of the other enzymes. The *ɛ*^2^ indicated no relationship.

**Table 4:** Enzymes determined in animals submitted to different treatments

Enzymes	CG	CHE	VBR	VBR + CHE	*P*	*ɛ*^2^
Amylase (μKat/l)	0.10 ± 0.00	0.10 ± 0.01	0.10 ± 0.01	0.10 ± 0.01	0.4557	0.1282
Lipase (μKat/l)	46.22 ± 25.32	46.04 ± 6.12	48.53 ± 6.67	48.32 ± 6.06	0.5098	0.1370
CK (μKat/l)	26.33 ± 8.52	25.78 ± 17.32	21.99 ± 6.27	19.65 ± 9.17	0.4456	0.1478
ALT (μKat/l)	2.18 ± 0.32	2.54 ± 1.59	2.08 ± 0.62	2.41 ± 0.84	0.5528	0.1103
AST (μKat/l)	1.20 ± 0.12	1.23 ± 0.26	1.24 ± 0.10	1.15 ± 0.15*	0.0209	0.5129
ALP (μKat/l)	1.82 ± 0.54	1.75 ± 0.44	1.67 ± 0.21	1.44 ± 0.32	0.0761	0.3616

*P<0.05. Values are shown as median ± S.D., *P, ɛ*^2^. Abbreviation: μKat, microkatal.

## Discussion

In animals, the investigations performed with the association between some substances and WBV [12,13,32] have stimulated our investigation. In the current study, effect of the association between an aqueous extract of *C. ambrosioides* and WBV on some physiological parameters such as body mass, feed intake, stool consistency, and biomarkers plasma concentrations were assessed for 6 weeks. There was a significant increase in the feed intake of the animals submitted to WBV (VBR) in comparison with the other groups. The levels of AST due to the simultaneous treatment (VBR + CHE) group decreased in comparison with other groups. No differences in the body mass and stool consistency were found amongst the groups. The increase in the feed intake might be related to the frequency of the mechanical vibration used in the WBV exercises. Furthermore, the decrease in the levels of AST in VBR + CHE group would be related with the long-term exposure of WBV (6 weeks).

Considering the body mass, no difference was found in 6 weeks amongst the groups: CHE, VBR, and VBR + CHE (Table 1). In relation to the WBV, Huang et al. [33] demonstrated that in WBV with frequencies of 5.6 and 13 Hz, the body weight of obese mice did not differ along 6 weeks. Otherwise, (i) Maddalozzo et al. [34] analyzed the body mass in healthy mature rats submitted to vibration (30–50 Hz) and observed that along 12 weeks, animals of the vibration group weighed less than the CG, and (ii) Naghii et al. [13] reported a significant increase over 8 weeks in the body weight of rats submitted to vibration (10–50 Hz) and treated with some substances (calcium, vitamin D, and boron), when compared with CG.

The 6-week vibration training resulted in significant increase (*P*<0.001) in feed intake (Figure 3) in the animals of the VBR group. This result is in accordance with Frederico et al. [11] that observed an increase in feed intake of Wistar rats submitted to the WBV at the same frequency (50 Hz). In a different way, Naghii et al. [13] evaluated the feed intake in healthy rats submitted to mechanical vibrations with frequencies of 10–50 Hz, and exposure time of 25–60 min, and no differences were observed in the amount of feed intake between the CG and the treatment groups. Lin et al. [35] analyzed the feed intake in middle-aged mice with 4-weeks WBV training regimen (5.6 and 13 Hz, exposure time of 15 min) and did not find any difference. While, in the current study using WBV with 50 Hz and exposure time of 5 min, an increase (*P*<0.05) in the feed intake was found in VBR group in comparison with CG, CHE, and VBR + CHE (Figure 3). This finding may be related with the biomechanical parameters such as frequency and the long-term exposure used during the WBV exercise. Moreover, our findings could be associated with the results reported by Wang and Kerrick [36] which verified that applying vibration to intact or skinned single-fiber preparations would incur specific increases in ATP turnover.

According to the findings with *C. ambrosioides*, the rats of the CHE group showed a decrease (*P*<0.001) in feed intake in comparison with the other groups, showing that the *C. ambrosioides* might be influencing a low consumption of feed. Kato et al. [37] also observed, in lambs, a significantly less intake in the group containing *C. ambrosioides* in feed, when compared with the CG. It was also expected on our investigation an alteration in VBR + CHE group, once the differences were observed separately in the groups VBR and CHE as shown in Figure 3. However, no differences were observed in the consumption of the VBR + CHE group when compared with other groups. This suggests that the association between mechanical vibration and the extract does not affect the appetite, that possibly may have occurred due the opposite effects showed by both agents.

Some studies have described that long-term exposure to WBV produces gastrointestinal disorders [38,39]. In physiological situations, the characterization of feces may be quite useful and the stool consistency is a possible method to observe these disorders [40]. In the current investigation, no statistical differences in stool consistency were observed amongst the groups. Considering the protocols using WBV exercise (Table 2), the long-term exposure to mechanical vibration was not capable of interfering with the steps in the stool formation.

The measurement of the concentration of blood biomarkers can help to assess the functions of internal organs. According to biochemical analysis, no changes were found in the plasma concentrations of various biomarkers evaluated in the current investigation in various treatments (Table 3). However, using a different protocol with WBV with 12 Hz (10 days), Frederico et al. [32] have verified an alteration in the concentration of cholesterol, triglyceride, and bilirubin. Lin et al. [35], used WBV with 5.6 and 13 Hz (4 weeks), observed a decrease in creatinine levels. Probably the effect of the WBV on the concentration of biomarkers would be related to the frequency used in WBV and/or the time of the exposition.

Concerning only the effect of *C. ambrosioides*, da Silva et al. [5] have identified a decrease in creatinine levels of serum after 15 days of administration of *C. ambrosioides* (1.0 g/kg), although, in the concentration used in our study (0.05 g/kg), no alteration was found in the plasma concentration of this biomarker.

The measurement of the plasma concentration of some enzymes can help to verify some information about the metabolism in some organs. Brancaccio et al. [41] suggest that CK can be used as a biomarker for prognosis of muscle injury. Aminotransferase serum levels (AST and ALT) are released from hepatocytes and by plasma clearance [42], and Bolanle et al. [43] have considered that AST and ALT are important prognostic indicators of hepatic injury.

In the current study, the effect of 6-week WBV (50 Hz) on enzymatic activities in rats treated with *C. ambrosioides* (Table 4) showed only a decrease in the levels of AST in the VBR + CHE group compared with other groups. The concentrations of CK, amylase, and lipase were not altered in our study (Table 4). Huang et al. [33] reported a significant reduction in serum levels of AST, ALT, and CK in obese mice submitted to WBV exercise (13 Hz). Lin et al. [35] also observed a decrease in CK levels in middle-aged mice exposed to WBV (5.6 and 13 Hz). It is relevant to consider that in our study the frequency used was 50 Hz, while Huang et al. [33] utilized 13 Hz, and Lin et al. [35] used 5.6 and 13 Hz. The utilization of different frequencies by the authors could justify the variability of results.

Kato et al. [37] treated lambs with *C. ambrosioides* oil that showed a significant increase in levels of AST; however, in the current study, alterations in the plasma level of this enzyme were not found in a single treatment with the medicinal plant. Nevertheless, a decrease in the concentration of AST was found with the joint treatment involving *C. ambrosioides* and WBV.

Putting together the findings, treatments with WBV and/or *C. ambrosioides* would be not capable of inducing injuries in the liver, as evaluated with ALT and in the muscle, as assessed with CK.

A limitation of the present study was that only one frequency (50 Hz) was used, as well as a single concentration of the *C. ambrosioides*.

In conclusion, based on the current investigation, the findings suggest that WBV may alter the physiological parameters, as the alteration in the feed intake without directly affecting the body mass. Moreover, WBV in association with *C. ambrosioides* causes alteration in the enzymatic activity of AST.

## Abbreviations

ALT: alanine aminotransferase
AST: aspartate aminotransferase
CG: control group
CHE: treated with *Chenopodium ambrosioides* group
CK: creatine kinase
OVP: oscillating/vibratory platform
VBR: exposed to 50 Hz of mechanical vibration
VBR + CHE: treated with *C. ambrosioides* and exposed to 50 Hz mechanical vibration
WBV: whole body vibration
